# Spatial dimensions of telemedicine and abortion access: a qualitative study of women’s experiences

**DOI:** 10.1186/s12978-019-0759-9

**Published:** 2019-07-03

**Authors:** Katherine Ehrenreich, Cicely Marston

**Affiliations:** 1 London School of Hygiene and Tropical Medicine, Keppel St, Bloomsbury, London, WC1E 7HT UK; 2 Present address: Advancing New Standards in Reproductive Health (ANSIRH), University of California, San Francisco, 1330 Broadway, Ste 1100, Oakland, CA 94612 USA; 3 London School of Hygiene and Tropical Medicine, 15-17 Tavistock Place, London, WC1H 9SH UK

**Keywords:** Abortion, Telemedicine, Information visit, Waiting period, Spatial theory

## Abstract

**Background:**

Telemedicine may help women comply with onerous legislative requirements for accessing abortion services. In Utah, there are three mandatory steps: a state-mandated information visit, a 72-h waiting period, and finally the abortion procedure itself. We explored women’s experiences of using telemedicine for the first step: the information visit.

**Methods:**

We conducted 20 in-depth interviews with women recruited from Planned Parenthood Association of Utah in 2017 and analyzed them using iterative thematic techniques, using a framework based on Massey’s conceptualization of space as comprising temporal, material and social dimensions.

**Results:**

Temporal, material and social dimensions of women’s access to abortion services intertwined to reduce access and cause discomfort and inconvenience among women in our sample. The 72-h waiting period and travel distance were the key temporal and material barriers, while social dimensions included fear of social judgement, religious influence, and negative stereotyping about people who have abortions. Women described traveling long distances alone and risking excessive pain (e.g. denying pain medication in order to drive immediately after the procedure) to try to overcome these barriers.

**Conclusion:**

Using telemedicine helped patients reduce burdens created by policies requiring attendance at multiple appointments in a state with limited abortion services. Attending to spatial aspects of abortion provision helps identify how these different dimensions of abortion access interact to reduce access and impose undue burdens. Telemedicine can improve privacy, reduce travel expenses, and reduce other burdens for women seeking abortion care.

## Plain English summary

In the United States, state-based restrictions on abortion include requirements for information visits (where state-required information about abortion and pregnancy is read from a script, sometimes including inaccurate information) and waiting periods between attending information visits and receiving an abortion. These requirements can be burdensome for women, particularly those who live long distances from abortion services. The state of Utah requires an information visit followed by a 72-h waiting period before any abortion procedure, meaning two appointments are needed. In 2015, the Planned Parenthood Association of Utah began offering telemedicine so that women can complete information visits with a health care provider over video conference. In this paper we examine women’s experiences using telemedicine for information visits in Utah. We conducted 20 in-depth interviews in 2017 with women who used telemedicine to attend information visits in Utah, and examine spatial aspects of women’s experiences. These women’s narratives show how temporal, material and social dimensions of space intertwined to reduce abortion access, also causing women discomfort and inconvenience. The 72-h waiting period and travel distance were the key temporal and material barriers, while social dimensions included fear of social judgement, religious influence, and negative stereotyping about people who have abortions. Using telemedicine helped women reduce burdens created by policies requiring attendance at multiple appointments in a state with limited abortion services. Exploring these spatial aspects of abortion provision helps identify how these different dimensions of abortion access interact to reduce access and impose undue burdens.

## Background

Abortion, one of the most highly contested subjects in American politics, is integral to wider debates over healthcare, women’s rights, racial justice, and religious freedoms. While abortion access is protected by national legislation in the United States (U.S.), state-based policies to restrict access have become increasingly common.

In Utah, a Southwest state in the U.S., state legislation mandates a pre-abortion informed consent disclosure visit (or information visit) that is performed face to face, during which patients must be read a state-mandated script containing information on the risks of abortion and pregnancy, and inaccurate information on medication abortion reversal [1]. This is then followed by a mandatory 72-h waiting period between the information visit and the abortion itself [1]. These requirements mean that patients must arrange two separate appointments in order to terminate their pregnancy. Along with information visit and waiting period requirements, insurance policies and public funding for abortion are highly restricted in Utah [2]. The state additionally requires parental consent for minors accessing abortion [2]. These combined restrictions leave abortion difficult to access in Utah.

Only two clinics provide abortion services in Utah, both which are located in Salt Lake City [3]. One of these clinics is a Planned Parenthood Association of Utah (PPAU) affiliate. PPAU has an additional seven outlying clinics that offer in-person information visits, but do not provide abortion services. Patients may be provided information visits by independent physicians, yet few providers in Utah offer this service [4]. Despite the large size of the state (84,899 mile^2^), most of the seven outlying clinics are clustered within 50 miles of Salt Lake City. To help patients reduce the burden of attending two appointments, PPAU introduced telemedicine, the provision of health care at a distance using information and communication technology [5], as an option to fulfill the first appointment. Telemedicine for information visits involves a video conference in which a health care provider reads aloud the mandated script to the patient, using any device with video and internet access. The information visit does not imply any deeper therapeutic or professional counseling.

This study explores women’s experiences of accessing abortion care using telemedicine to fulfill the information visit requirement in Utah. We examine spatial aspects of abortion access to understand how telemedicine affects women’s experiences. We use Doreen Massey’s conceptualization of space to structure our analysis. We analyze women’s experiences accessing abortion in terms of the dimensions of space that Massey describes: material, temporal and social. For Massey, culture does not exist merely within the confines of physical borders, it shapes expectations of interactions with space.

Space is by its very nature full of power and symbolism, a complex web of relations and domination and subordination, of solidarity and co-operation. This aspect of space has been referred to elsewhere as a kind of power-geometry [6].

In this paper, we attend to the properties of spaces where women seek and receive abortion services as a way to understand the nuanced implications of telemedicine provision in the context of restrictive abortion laws.

We explore how access to abortion both shapes and is shaped by the material, temporal and social aspects of spaces, and their interrelationships. Our study explores women’s experiences of using telemedicine for information visits to fulfill abortion requirements, and acceptability of telemedicine as a way to provide this information: an area not examined previously.

### Telemedicine for abortion

Research on effectiveness and acceptability of telemedicine for abortion has focused largely on medication abortion (MA) [7–11]. In the U.S., telemedicine for MA allows patients in remote clinics without an abortion provider to consult with a physician over video and be prescribed MA in real time. Studies show that provision of MA via telemedicine versus face-to-face provision have comparable clinical outcomes, and low prevalence of adverse events [8, 10]. In states where physicians who provide abortions are scarce, telemedicine allows physicians to prescribe safe and legal MA to patients over long geographic distances [5, 7], reduces physician travel to outlying clinics [8], and aims to improve access to early abortion [8].

Introduction of telemedicine has been associated with positive outcomes elsewhere. For example, in Iowa, introduction of telemedicine was followed by a decrease in second-trimester abortions [8]. In Alaska, medication abortion via telemedicine was found to facilitate a more patient-centered approach to care, enabling patients to access care sooner, have more choice in procedure type, and be seen closer to their home [8]. Healthcare providers in Alaska also reported that telemedicine had minimal impact on the quality of provider-patient interaction, despite the visit taking place over video [7].

## Methods

In May – October 2017, we conducted 20 in-depth interviews for the Utah Telemedicine Evaluation Project (UTEP), a mixed methods study led by Advancing New Standards in Reproductive Health (ANSIRH) at the University of California, San Francisco (UCSF).^1^ Interviews were conducted by KE (author 1) and a member of research staff from ANSIRH. One interview was conducted in Spanish by an additional staff member from ANSIRH. Planned Parenthood staff invited patients to complete a self-administered online survey at the time of their information visit. The survey included questions on demographics, whether the participant attended their information visit in person or by telemedicine, and satisfaction with their telemedicine visit. We invited individuals to participate in an in-depth interview who were over age 18, spoke English or Spanish, who had used telemedicine, and who had given consent within the questionnaire to be invited for interview. Survey participants received $25 in gift cards to thank them for their participation, and in-depth interview participants received a further $50 in gift cards. Thirty-two survey participants indicated they were interested in an interview and were contacted. Twenty-two participants responded, of which one declined to participate and one was ineligible.

The in-depth interview asked women about their experiences using telemedicine to access abortion care, contextualizing this by asking them about their past health care; pregnancy experiences; abortion decision making, access and experiences; and their knowledge and opinions about abortion laws. We contacted eligible participants by phone or email up to three times, and conducted 20 interviews. Two interview participants had incorrectly completed the survey and had not in fact used telemedicine. This was not clear until after the interviews were underway. Because their interviews are relevant to other areas of this analysis, they are nevertheless included. Interviews lasted 30–75 min and were recorded and transcribed with consent. Participant 19’s interview was conducted in Spanish, with the transcript containing the English translation of what was said (there was no separate transcription in Spanish). Because translations are always open to interpretation, CM (author 2) listened to the audio in Spanish and added small corrections, amendments, and notes to aid interpretation.

We analyzed interviews using iterative thematic techniques. In this respect, analysis followed a grounded theory approach. We used deductive and inductive techniques with reference to spatial theory [6, 12]. Participants’ experiences using the telemedicine platform, including their motivations for choosing telemedicine and their perceptions of the provider-patient relationship, are explored in a separate article [13].

This study was approved by the London School of Hygiene and Tropical Medicine Research Ethics Committee, and the Institutional Review Board at the University of California, San Francisco.

## Findings

Eighteen of our interviewees used telemedicine to attend information visits, of whom 17 scheduled abortion appointments. One participant scheduled an abortion but her pregnancy ended in a miscarriage prior to the appointment. The remaining 16 participants traveled to Salt Lake City for abortion care after their telemedicine information visit, one of whom decided at the clinic to continue her pregnancy. The two participants who chose to continue their pregnancies were ambivalent about having abortions prior to their information visits.

Participants 19 and 20 did not use telemedicine. Participant 19 attended an in-clinic information visit at a PPAU affiliated clinic; and Participant 20 decided to avoid the 72-h waiting period by traveling to Nevada for her abortion and reported that she did not participate in any information session in Utah.

Table 1 shows participant characteristics. Those who opted for telemedicine lived a median distance of 133 miles from Planned Parenthood in Salt Lake City (range of 8–280 miles). The 72-h mandated waiting period translated into a median wait of 8 days (range of 5–17 days), typically due to lack of appointment availability that aligned with participants’ scheduling and travel needs. In Utah, patients are not permitted to schedule or reserve abortion appointments until they have completed an information visit. Among the 20 participants, seven traveled outside of their home states to access their abortions. Five participants had never been pregnant before; five participants had one previous pregnancy; seven participants had two to three previous pregnancies; and three participants had been pregnant five or more times.

**Table 1:** Summary of participant characteristics

Participant	Age	Race/Ethnicity*	Completed education*	Previous induced abortion(s)	Approximate distance from participant’s home to abortion clinic in Salt Lake City (miles)	Lived out of state	Days between telemedicine and scheduled abortion appointment	Pregnancy outcome
1	35–39	White	Professional or advanced degree	Yes	215	Yes	8	Surgical abortion
2	20–24	White	Some college	No	280	Yes	7	Surgical abortion
3	35–39	White	Some college	No	32	No	6	Medication abortion
4	25–29	Native American	High school degree or GED	No	272	Yes	N/A	Continued pregnancy
5	25–29	White Hispanic	College degree	No	250	No	8	Surgical abortion
6	25–29	White	College degree	No	250	No	8	Continued pregnancy
7	25–29	White Polynesian	Professional or advanced degree	No	125	No	11	Surgical abortion
8	25–29	White Hispanic	College degree	Yes	N/A^4^	Yes	12	Surgical abortion
9	18–19	White	High school degree or GED	No	106	No	7	Medication abortion
10	20–24	White Hispanic	Less than high school	No	10	No	8	Surgical abortion
11	25–29	Black or African American	Some college	No	170	Yes	15	Surgical abortion
12	35–39	White	Professional or advanced degree	No	217	Yes	17	Surgical abortion
13	25–29	Asian American or Pacific Islander	College degree	No	9	No	8	Medication abortion
14	25–29	Black or African American	High school degree or GED	No	32	No	5	Surgical abortion
15	40–44	White	College degree	No	9	No	8	Miscarriage
16	35–39	White	Less than high school	Yes	11	No	10	Surgical abortion
17	18–19	White Hispanic	Some college	No	253	No	16	Surgical abortion
18	30–34	White	College degree	No	26	No	8	Medication abortion
19	25–29	White Hispanic	N/A**	No	51	No	N/A	Surgical abortion
20	35–39	White	College degree	No	Traveled 480 miles to Nevada	Yes	N/A	Surgical abortion

*Pre-specified survey categories for participants to choose from

** Missing data

^4^Participant 8’s travel distance is unclear because she was traveling on a cross-country road trip at the time of her pregnancy, and rerouted her trip in order to access abortion care

### Material and temporal dimensions of abortion access

The most evident temporal dimension of abortion access for our participants was the mandatory 72-h waiting period. Women commonly reported they were unaware of this waiting period law until they called the clinic to schedule an abortion. Some women sought options in other states to avoid this restriction, but ultimately completed the waiting period in Utah.

Participant 12, who drove 5 hrs to Salt Lake City from Idaho, explained that she could have driven 2 hrs to access abortion services in Idaho, but Idaho state policies would have required her to attend more appointments. She chose to drive to Salt Lake City because there she was only required to physically attend one appointment with the option to attend the information visit via telemedicine. Participant 20 told us she began experiencing signs of pregnancy while recovering from being raped. After confirming her pregnancy, she called the clinic and learned about the 72-h waiting period.

Every time I had some sort of obnoxious symptom [of pregnancy], it just made me relive the rape. […] [So] I couldn't stand to wait another week. Because even though it was the 72-hour hold […] the availability [was] so far out that it was going to be a couple more weeks before I was able to get in. And I couldn't wait that long.

She found an alternative option with a clinic in Nevada, which was available within 48 h.

I wish I had known about the timing, or the 72-hour thing. I probably would have [called Planned Parenthood] much sooner. […] I had a feeling long before I got the actual test. I just, I don't know. I buried my head in the sand. I didn't want it to be true […]. You know, ignore it long enough, maybe it will go away, which I know is ridiculous for an educated [35-39] year old mother of two. But it was just, you know, it was too much to deal with. The rape itself was too much to deal with, and then having this on top of it made it really too much to deal with. (Participant 20, traveled 480 miles, surgical abortion)

Other temporal dimensions of abortion access were often interrelated with the material, particularly the lack of nearby facilities for many participants. Participants commonly explained that abortion access, especially in rural areas, was scarce or non-existent. They reported traveling long distances to access abortion care, and incurred costs associated with travel and child care. Most women who traveled from rural areas said they found it frustrating to have to travel to the city for abortion care when they would normally travel for approximately 15 min to access other health care.

Most said they had opted for telemedicine largely because of travel distance.

I live about five hours from that location, so if that wasn’t available to me I would have to go make a ten-hour drive just to have that little fifteen-minute meeting, in person. […] And then come back again […] for the actual procedure. It would have been a big waste, a big, big time-consuming event, if I had to actually go travel to see somebody rather than do [the information visit] over the internet. (Participant 5, traveled 250 miles, surgical abortion)

When we asked participants if there was anything they wished they could tell state politicians, most of them commented on access.

Interviewer: What would you want lawmakers to know about abortion, or your experience?

Respondent: To make it more accessible, in, like, rural areas, so, if that’s possible. I don’t know that that’s a lawmaker’s job, though. (Participant 1, traveled 215 miles, surgical abortion)

The location of the clinic appeared to affect some women’s experiences accessing abortion care, reporting it was difficult to find. Participant 18 described the clinic as being “hidden away.” In her narrative, the location of the clinic problematically reinforced the sense that abortion was “frowned upon”:

It feels, like, hidden away. And I think that might be because of the general perception of Planned Parenthood and abortion […]. So, it kind of encourages that negative perception, I think. […] As in, ‘We’re going to hide this very valid medical service away from public view because it’s frowned upon by general public who have this moral high ground that they feel a pregnancy is a gift from god.’ […] It feels like it’s tucked away because, almost like a shame of it, rather than in a big, bright medical institution, you know, where you’d go for any other medical procedure. (Participant 18, traveled 26 miles, medication abortion)

Participant 2, however, seemed to prefer that the clinic was “hidden”:

It was in a building where there were multiple different businesses and offices, so I think that kind of masks it a little bit. It was hidden. Which is, I mean, you know, you don't really want a big old building with ‘Planned Parenthood’ and everything written all over it. Especially in Utah. (Participant 2, traveled 280 miles, surgical abortion)

### Social dimensions

Women’s narratives consistently indicated they expected others to hold negative views and stereotypes about women who have abortions. Women commonly described adjusting their travel plans or appointments because of their fear of being judged. Several women described telemedicine as having made them more comfortable, particularly those who felt they would face social repercussions if seen at their local clinics; and were relieved they had the choice to complete the appointment from their homes. Participant 10 had failed to attend her first scheduled in-person information visit, which she had made before she learned about the option to use telemedicine.

The first [information] appointment that I had, I didn't have the money for, and I also just kind of chickened out because it was right next to where I live. I know a lot of people in the area, and I really just didn't want to like, show up at a class where I was going to know people and like, have people know what I was going through. So, I called back after I had already missed that appointment […], and then the lady told me about the app that you could do the visit on from home. (Participant 10, traveled 10 miles, surgical abortion)

Several other participants also said they used telemedicine because of privacy. This was of particular interest to Participant 2, who said she chose to not access abortion services in her hometown because she was worried about being seen at the clinic.

You can do [the information visit via telemedicine] in your own privacy. You don't have to worry about, you know, going to the parking lot, walking in, and having, you know, Miss Susan the neighbor walking by with her dog and seeing you walk in. (Participant 2, traveled 280 miles, surgical abortion)

Many women told us they traveled alone over long distances to access abortion services and had avoided asking for support because they worried about social repercussions. The temporal, material and social dimensions of access to abortion services intertwine to reduce access and cause discomfort and inconvenience. Participant 2, for instance, said she did not ask her sister to accompany her on the 280-mile drive to Salt Lake City because she did not want to “put anyone else in that kind of situation.” She also said she did not feel comfortable asking another sister to accompany her, even though the second sister already lived in Salt Lake City. When Participant 2 arrived at the clinic, however, she said she felt lonely seeing other women supported by partners. She told us that she was determined to drive home on the day of the procedure to avoid missing an extra day of work. Because she did not have anyone to drive her, she deliberately limited her intake of pain medication so that she could drive herself home:

I knew going in […] I cannot be given pain killers that cannot let me drive after. […] I'm here alone and I have no other choice. And you have to give me the least amount you can give me. […] I can handle the pain. The pain's not unbearable. I have to be able to drive. (Participant 2, traveled 280 miles, surgical abortion)

Women commonly referred to religious influences when they talked about their experiences of accessing abortion.

Well, when I had my first pregnancy, I was still LDS [i.e. belonged to the Church of Latter Day Saints]. I wanted an abortion really bad, but I couldn't do it because […] I was still in the church […]. We were taught that if we had an abortion, we're going to hell, so I couldn't do it. […] But, I mean, going back, if I could go back without knowing what it's like to have a child, I might still do it if I didn't believe what I did at the time. (Participant 13, traveled 9 miles, medication abortion)

Participant 2 described at length how she thought her religious upbringing affected her abortion experience:

And as soon as they turned [on the vacuum for the procedure] it was very dehumanizing. […] I felt all the guilt all of a sudden. […] Because it wasn't the physical pain that was breaking me, it was the loneliness. […] Which I don't even – I'm not a religious person. I grew up in a religion, of course, in a Christian religion, but I'm not religious. […] I'm not exactly sure if it was maybe the guilt rising up from, you know, my past, what I was raised in. (Participant 2, traveled 280 miles, surgical abortion)

Religion also appeared to influence some women’s interactions with medical professionals, or while receiving medical care. Some described being approached by nuns or religious volunteers in hospitals during previous pregnancies, which they said made them uncomfortable.

Participant 7 described how her doctor, a private practitioner whom she told us had performed a tubal ligation on her that had failed,^2^ tried to convince her to carry her subsequent pregnancy to term, despite the fact she had a medical condition that would endanger her life if she became pregnant again:

When I was talking to [the doctor], she was letting, like, more religious views come in before medical views. […] She said, “All things happen for a reason,” and, “You got pregnant for a reason.” […] I had told her that I didn’t want to try and carry a baby to term because I knew that it would put my life at risk and that, by doing that, that’s putting my two children’s [lives] at risk. […] And at that point, she just said, “Well, I don't deal with terminations and you’ll have to seek other options.” (Participant 7, traveled 125 miles, surgical abortion)

Women often said that they lacked access to information about contraception and reproductive health in general because of religious influence among state politicians. Some women credited their lack of reproductive health knowledge to Utah’s abstinence-based sex education policy.^3^ Participant 19 said that before her own experience of having an abortion, she was “one of those people who was against abortion” for religious reasons. She said that becoming informed about abortion changed her perspective, and that she wished other women could be informed, too. She went on to say:

Like in this state, family planning is awful. It’s expensive, it’s scarce. So, there are many women who have loads of children because there’s no form of family planning [here]. So, there aren’t any options [for them]. So, many women just keep on having children. Like me, I wanted to have surgery [sterilization] when my son was born, but [the hospital] wouldn’t do the surgery because of my age, because I’m very young. And I think that part of what is causing problems here in Utah is the Mormon religion. I don’t have anything against it or anything, but yes it has a big impact because the religion means that contraception is very, very scarce. […] [Having an abortion] doesn’t make you feel guilty. Just the opposite, it makes you feel good because the economy is really, really bad right now, so to bring a child into the world to suffer isn’t right. (Participant 19, traveled 51 miles, surgical abortion)

Many women told us about the ways they believe lawmakers hold inaccurate stereotypes about women who have abortions, and described their frustrations with the legal restrictions around abortion.

I really want [lawmakers] to be compassionate about the laws that they make. Compassionate for the woman who has to make this decision that affects the rest of her life, and the lives of the people around her. […] Or whatever [her] situation might be that they have no fucking clue about. […] That's why women are in this situation, it’s because they need help, and they need someone to show them compassion. (Participant 6, traveled 250 miles, continued pregnancy)

Interviewees often contrasted their own experiences with what they saw as inaccurate stereotypes about abortion and women who have abortions:

I feel like people think that people are using abortion as, like, a form of birth control. […] If you've been through one and you've had one, like, you're not using that as a form of birth control. It's definitely, like, traumatic. […] I feel like people just think that, like, that people don't put any thought into it. […] And it's definitely something that you think about, like, a lot. […] Because it's not like it's cheap by any means. And then on top of the expense, it's, in Utah at least, […] it's not an easy thing to go through because you have your wait time and you have to have your consent and things like that, which the video consent did, like, make it a little bit easier. But it's nothing that makes it so much easier that I feel like people wouldn't be on birth control because of it. (Participant 7, traveled 125, surgical abortion)

While our interviewees were familiar with the problems associated with negatively stereotyping people who have abortions, many nevertheless often referred to other (hypothetical) women for whom it could be necessary to put barriers in place, in order to deter those other women from recklessly using abortion services. For instance, while they all said that the 72-h waiting period had made little or no difference to their own abortion decision making, and had had a negative impact in terms of making the procedure more inconvenient and costly, many interviewees also said the waiting period might nevertheless be helpful for other women. One participant implied that unrestricted abortion might lead to more abortions (that other women would have) despite telling us that she made her own decision on the basis of careful assessment of her situation.

Like mine? It was a good thing because I don’t think we were ready for that. [But] if it’s easier [i.e. abortion], then people will just be getting pregnant every day […]. I don’t think that would be good. (Participant 11, traveled 170 miles, surgical abortion)

Participant 15 scheduled an abortion because of her medical history and the associated health risks a pregnancy would pose to her and the fetus. Her pregnancy ended in a miscarriage three days prior to her scheduled abortion appointment. She said that having an abortion would have been the right decision for her, but that the “average healthy woman” was selfish for having an abortion, citing rumors of other women (“nobody I know”) who have multiple abortions after “just not caring” (i.e. not caring enough to use birth control).

Respondent: You know, like these young kids and stuff, especially like if they've been raped or whatever, like that, it’s a completely different situation. I'm talking about grown adults that just make bad decisions.

Interviewer: When you say “bad decision,” do you mean like, not using birth control?

Respondent: Right. Yeah, just not caring. And like I said, I've heard stories out there where, you know, they just don't use anything at all, and they get pregnant. […] There's people here that have five, six, 10 abortions. It's disgusting. I mean that's just so wrong.

Interviewer: You've heard through the grapevine people who have done that?

Respondent: Yes, exactly. It's nobody that I know.

Later in the interview, she continues:

I think the [information visit] would be beneficial and the waiting period, and giving them all options. […] Like straight-out asking them why they're making that decision. Like why? If it's just because you're selfish and you don't want a baby? You don't want to raise a kid, you know, for the next 18 years until they’re adult and out of the house? Well, then give it up for adoption if that's your only reason. It's nine months out of your life. You know? And I think a lot of people do it just because they're selfish. […] They don't want to take on responsibilities. You know, if they're drinkers or partiers or, you know what I mean? They travel a lot. Or all this other stuff. Or if they work too much, you know. All these excuses that I believe are completely irrelevant, that I just think are selfish. (Participant 15, miscarriage)

Participant 2, who traveled 280 miles alone and described an extensive decision process, also worried about other women “taking advantage” of accessible abortion provision.

I mean it would be nicer and it probably would be easier and safer […] to have these clinics in areas where, not necessarily have easy access, but easier access to. […] I think they're kind of like Walmarts. The more you have of them the more people kind of take advantage and, you know, go to Walmart just because they can go to Walmart. […] I feel like if it's too accessible, it becomes a little, it becomes more of a normal thing. […] I can see it breaking people. The guilt. (Participant 2, traveled 280 miles, surgical abortion)

Participants did not provide any real life examples to support their idea that irresponsible women of this type might exist.

## Discussion

Attending to spatial aspects of abortion provision shows how these dimensions of abortion access interact to create additional risks and burdens on women who seek care. For instance, fear of social judgement affects how women navigate physical spaces to access abortion services. The ways in which social barriers make material access difficulties more pronounced and increase discomfort and pain are illustrated by Participant 2, who limited her intake of pain medication so she could drive a long distance after her procedure in order to get home. Participant 2 had to use her car to overcome the material dimensions of access, but her reason for not having someone else drive was that she did not want to ask one of her sisters to accompany her (i.e. a social barrier). Participant 2 had not wanted to use abortion services in her hometown because she wished to avoid being seen at the clinic, yet her next closest option was 280 miles away in another state, owing to the lack of clinics in the region. The social implications of local access contributed to her risking excessive pain because she believed this was her only option in order to have an abortion and be able to drive safely. In this way, making abortion hard to access may put women at unpredictable yet entirely avoidable risk of excessive pain and negative social consequences.

Participants’ expectations of society’s negative stereotypes of people who have abortions also influenced the ways they experienced accessing abortion services. For example, Participants 2 and 18 perceived Planned Parenthood’s “hidden” location differently; while both understood that it was a response to social perceptions of abortion, one said it reinforced negative perceptions (Participant 18) and the other was grateful for it (Participant 2). Whereas Participant 18 lived in an urban area and expressed throughout her narrative that she was “feminist” and “pro-choice,” Participant 2 described in detail her religious and conservative upbringing, highlighting how detrimental it would be to her if her community found out about her abortion. Social dimensions of abortion provision can both shape and be shaped by the material dimensions and vice versa.

Participants with a lack of financial resources, further travel distances to access abortion care, and who experienced a heightened fear of social judgement appeared to be disproportionately affected by the requirement for the information session and the 72-h waiting period. For example, these participants commonly said they postponed their abortions until they saved enough money for the procedure or could coordinate travel without disclosing their abortions to others, which meant they had more expensive procedures at a later stage of gestation. While we did not collect information about income status directly, it appeared that women who described having fewer economic resources relative to our sample as a whole were disproportionately disadvantaged by the information visit and waiting period requirements.

The ways women presented themselves in relation to negative social perceptions about abortion were diverse. Some emphasized that all women should be trusted to make these decisions, while others expressed that some women (although not themselves) may find the policies helpful in making their decision. The women who expressed ideas that the restrictive laws might be useful provided hypothetical examples only, and did not refer to their own lived experiences. This finding aligns with previous research that women may endorse the utility of abortion regulations for other women, but not themselves (14). The stereotypes women rejected when describing their own experiences are evidently so embedded in ideas about abortion access that many of the women reiterated the same negative stereotypes when talking about the hypothetical other women who seek abortion. By doing this, intentionally or otherwise, they may help to perpetuate these same stereotypes. Previous studies have found that anti-choice women commonly present to abortion clinics [14, 15]. While none identified themselves as anti-choice in their interviews, it is possible that some of our participants may have identified as such, either formerly or at the time of the interview.

It could be that when asked to reflect on the function of restrictive laws, the existence of these laws may lead individuals to believe that other women in society benefit from the legislation, despite their own experiences that often exemplified the opposite. In this way, the legal framework constructing abortion access helps shape women’s expectations of other women in social spaces. In this way, several participants both perpetuated and rejected social expectations and stereotypes about abortion.

We explored participants’ experiences using telemedicine to attend information visits through qualitative methods, and our study is limited in that we cannot compare the experiences between patients who attended this appointment by telemedicine and in person. Participants volunteered to be a part of the study, which may have resulted in self-selection bias. In common with most qualitative studies, we did not collect numerical data, so did not use a statistical sample, and any numbers or proportions are therefore not statistically generalizable. We cannot know for sure whether our findings would be transferrable to other settings, however we have no particular reason to doubt that they would be useful elsewhere.

## Conclusion

Our participants’ experiences align with previous studies demonstrating how restrictive state-based abortion policies increase burdens of travel, childcare expenses, and social judgement; disadvantages which disproportionately affect low-income women [16–19]. Telemedicine was a highly acceptable way to attend information visits, and reduced burdens experienced among the women in our study to access this appointment. In alignment with previous evidence on the benefits and patient acceptability of telemedicine provision of MA [7, 9–11], the use of telemedicine for information visits where these visits are required may lend to models where multiple steps of patient-provider interactions for abortion can occur via telemedicine, when appropriate. While telemedicine does not eliminate the burdens associated with restricted abortion access, particularly for women who live long distances from their nearest abortion provider, it moved the appointment from the clinic to women’s preferred private spaces, and by doing so, helped to mitigate some of the burdens imposed by policies requiring attendance at multiple appointments for abortion care. Attending to the spatial dimensions of abortion access helps to identify the barriers women face when attending multiple appointments for abortion, and how these burdens can interact to leave abortion patients socially and financially vulnerable.

## Data Availability

The data that support the findings of this study are available from UCSF. Restrictions apply to the availability of these data, which were used under license for the current study, and are not publicly available. Data are however available from the authors upon reasonable request and with permission of UCSF and the study’s Principal Investigator at ANSIRH.

## Footnotes

1 ANSIRH is a collaborative research group at the University of California, San Francisco’s Bixby Center for Global Reproductive Health

2 From the narrative it is unclear whether the tubal ligation procedure failed, or whether other factors were in play, e.g. that that provider had not excluded pregnancy before the ligation procedure.

3 Utah public schools require abstinence-based sex education curriculum, where “teachers are required to present a strong abstinence message” [20].
